# Lack of an Association between Neutrophil-to-Lymphocyte Ratio and PSA Failure of Prostate Cancer Patients Who Underwent Radical Prostatectomy

**DOI:** 10.1155/2016/6197353

**Published:** 2016-04-20

**Authors:** Yoko Maeda, Takashi Kawahara, Mitsuyuki Koizumi, Hiroki Ito, Yohei Kumano, Mari Ohtaka, Takuya Kondo, Taku Mochizuki, Yusuke Hattori, Jun-ichi Teranishi, Yasushi Yumura, Yasuhide Miyoshi, Masahiro Yao, Hiroshi Miyamoto, Hiroji Uemura

**Affiliations:** ^1^Departments of Urology and Renal Transportation, Yokohama City University Medical Center, Yokohama 2320024, Japan; ^2^Department of Urology, Yokohama City University, Graduate School of Medicine, Yokohama 2360004, Japan; ^3^Departments of Pathology and Urology, Johns Hopkins University School of Medicine, Baltimore, MD 21287, USA

## Abstract

*Introduction*. The neutrophil-to-lymphocyte ratio (NLR), which can be easily calculated from routine complete blood counts of the peripheral blood, has been suggested to serve as a prognostic factor for some solid malignancies. In the present study, we aimed to determine the relationship between NLR in prostate cancer patients undergoing radical prostatectomy (RP) and their prognosis.* Materials and Methods*. We assessed NLR in 73 men (patients) who received RP for their prostate cancer. We also performed immunohistochemistry for CD8 and CD66b in a separate set of RP specimens.* Results*. The median NLR in the 73 patients was 1.85. There were no significant correlations of NLR with tumor grade (*p* = 0.834), pathological T stage (*p* = 0.082), lymph node metastasis (*p* = 0.062), or resection margin status (*p* = 0.772). Based on the area under the receiver operator characteristic curve (AUROC) to predict biochemical recurrence after RP, potential NLR cut-off point was determined to be 2.88 or 3.88. However, both of these cut-off points did not precisely predict the prognosis. There were no statistically significant differences in the number of CD66b-positive neutrophils or CD8-positive lymphocytes between stromal tissues adjacent to cancer glands and stromal tissues away from cancer glands and between different grades or stages of tumors.* Conclusions*. There was no association between NLR and biochemical failure after prostatectomy.

## 1. Introduction

The neutrophil-to-lymphocyte ratio (NLR) can be easily calculated from routine complete blood counts (CBCs) in the peripheral blood. The NLR has been suggested to be not only a predictor of the systemic inflammatory response in critical care patients, but also a prognosticator for some solid malignancies including prostate cancer [1, 2].

Prostate-specific antigen (PSA), also known as human kallikrein 3, has been widely used for the early detection of prostate cancer as well as monitoring of its treatment. However, nonmalignant conditions, in particular benign prostate hyperplasia and acute prostatitis, often raise the serum PSA level, which complicates the diagnosis of prostate cancer using the PSA measurement alone [3, 4]. We previously described the effectiveness of the NLR for predicting prostate cancer in men undergoing prostate biopsy by showing that the NLR was significantly higher in the prostate cancer group compared with the noncancer group [5]. We also confirmed the usefulness of the NLR for predicting the prognosis of prostate cancer patients who presented with metastatic disease and who underwent docetaxel chemotherapy.

In the present study, we aimed to determine whether the NLR served as a prognostic factor in prostate cancer patients undergoing radical prostatectomy (RP).

## 2. Materials and Methods

### 2.1. Patients

The NLR was determined in a total of 73 men who subsequently underwent RP at our institution from 2011 to 2014 for their prostate cancer. None of these patients received hormonal therapy or other anticancer treatments preoperatively or postoperatively prior to biochemical recurrence. Biochemical recurrence was defined as a single PSA of ≥0.2 ng/mL. The institutional review board of Yokohama City University Medical Center approved this study.

### 2.2. Clinical and Laboratory Assessments

The NLR was calculated using the neutrophil and lymphocyte counts via CBCs obtained before RP. To determine the differences in the surgical technique, all RPs were performed or supervised by one expert surgeon (Yasuhide Miyoshi). Tumor grade of the RP specimens was determined according to the ISUP consensus on Gleason grading [6].

### 2.3. Prostate Tissue Microarray (TMA) and Immunohistochemistry (IHC)

We retrieved separate 51 RP specimens obtained at Yokohama City University Hospital (Yokohama, Japan). Appropriate approval from the institutional review board at our institution was obtained before the construction and use of the TMA. This was constructed from each representative lesion (benign and carcinoma). The clinicopathologic characteristics of these 51 patients are summarized in Table 2.

IHC was performed on the sections (5 *µ*m thick) from the prostate TMA, as described previously [7], using a primary antibody to CD66b (clone G10F5, diluted at 1 : 200, BD Biosciences, San Jose, CA, USA) or CD8 (clone C8/144B, diluted at 1 : 100, DAKO Corporation, Carpinteria, CA, USA). The slides were examined by a single pathologist (Hiroshi Miyamoto) blinded to the sample identity. The total numbers of CD66b-positive and CD8-positive cells were counted in each TMA core.

### 2.4. Statistical Analyses

The patients' characteristics were analyzed using the Mann-Whitney *U* test, chi-square test, and one-factor ANOVA test. The NLR cut-off value was evaluated by the AUROC curve. The Kaplan-Meier product limit estimator was used to estimate the recurrence-free survival after RP. A log-rank test was used for comparison. All these statistical analyses were performed using the Graph Pad Prism software program (Graph Pad Software, La Jolla, CA, USA). Statistical significance was determined to exist at *p* < 0.05.

## 3. Results

### 3.1. The NLR Values Were Not Correlated with the Pathological Status

A total of 73 patients underwent RP with variable NLRs. The median calculative NLR was 1.85. The clinicopathologic characteristics of these patients are summarized in Table 1.

**Figure 1:**
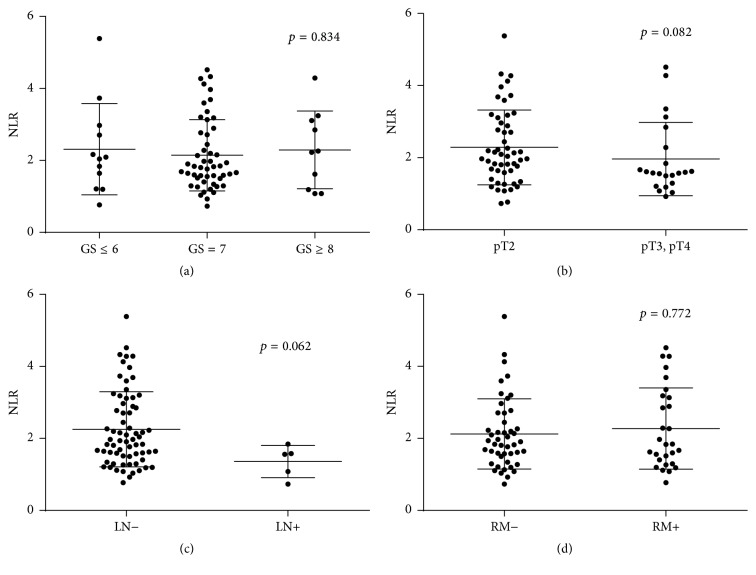
Comparison of the NLR with each prognostic factor, including (a) Gleason score, (b) pathological T stage, (c) lymph node metastasis, or (d) surgical margin status.

The NLR was not significantly correlated with histopathological features, including Gleason score (GS) (≤6 versus 7 versus ≥8, *p* = 0.834), pathological T stage (pT2 versus ≥pT3, *p* = 0.082), lymph node metastasis (negative versus positive, *p* = 0.062), or surgical margin status (negative versus positive, *p* = 0.772) (Figure 1).

### 3.2. The NLR Values Were Not Correlated with PSA Failure

**Figure 2:**
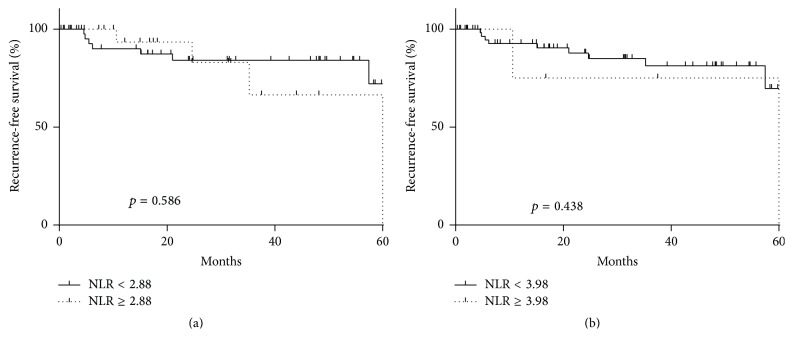
The recurrence-free survival according to the NLR.

Based on the AUROC curve, potential NLR cut-off point was 2.88 or 3.88 to predict PSA failure (AUC: 0.5092). The patients include 55 in low NLR group and 18 in high NLR group (NLR cut-off: 2.88). And median PSA recurrence-free survival was 63.8 months. However, neither of these cut-off points precisely predicted PSA recurrence after RP (Figure 2).

### 3.3. Infiltrating Neutrophils and Lymphocytes in Prostate Cancer Specimens

**Figure 3:**
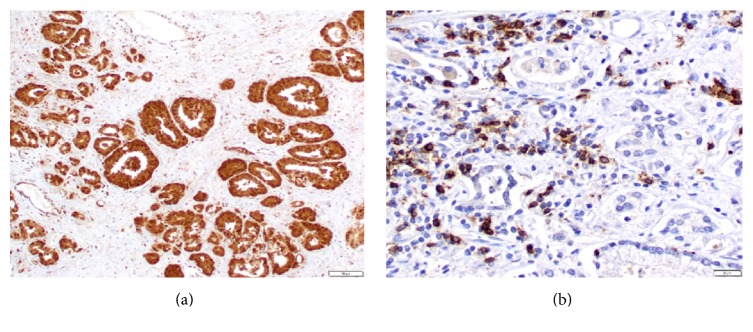
Immunohistochemical staining for (a) C66b and (b) CD8.

**Figure 4:**
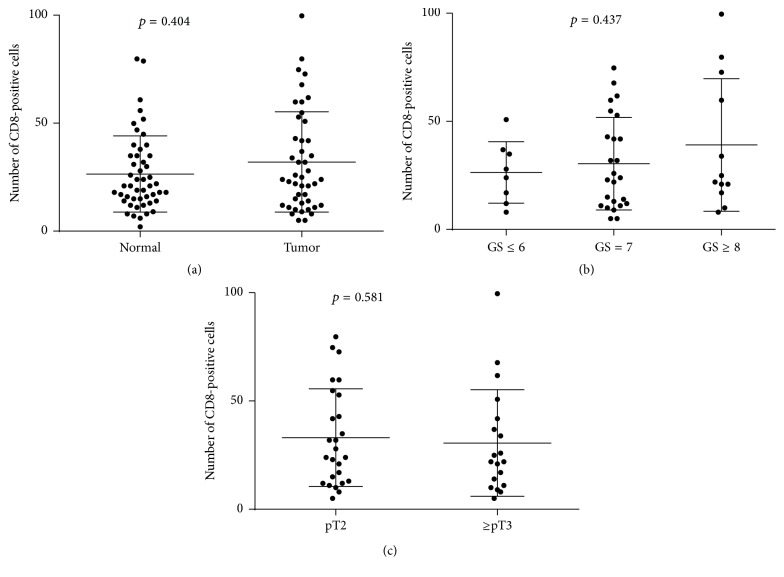
Number of CD8-positive cells in (a) normal and tumor tissues, (b) different GS, and (c) different pathological T stage.

Infiltrating CD66b-positive cells in the stroma were observed only in a few cases, while tumor cells were immunoreactive for CD66b in several cases (Figure 3(a)). Therefore, we analyzed the relationship between the number of infiltrating CD8-positive lymphocytes (Figure 3(b)) and clinicopathological features of prostate cancer. The number of CD8-positive cells in the stroma adjacent to the tumors was not significantly higher than that in the stroma around nonneoplastic prostate (*p* = 0.404; Figure 4(a)). In addition, there were no significant correlations between the number of CD8-positive lymphocytes and tumor grade (*p* = 0.437; Figure 4(b)) or pathological T stage (*p* = 0.581; Figure 4(c)).

## 4. Discussion

There is increasing evidence correlating the presence of systemic inflammation with a poorer cancer-specific survival in patients with several solid tumors, such as colorectal carcinoma [8–14]. Moreover, nonsteroidal anti-inflammatory medications have been suggested to reduce the risk of developing prostate cancer, implying a critical correlation between inflammation and prostate carcinogenesis [8, 9]. It has previously been demonstrated that the presence of an inflammatory response can be determined by both the expression of C-reactive protein and/or an elevation in the NLR [10, 15, 16]. In particular, the latter has been associated with a poorer prognosis in patients with prostate cancer [17].

Biochemical recurrence after RP has been associated with multiple factors, including the preoperative PSA level, the pathological stage, the GS of RP specimen, and the surgical margin status [18, 19]. Although our study confirmed these observations, we did not find strong associations between the NLR and any prognostic or clinicopathological factors. Regarding the NLR for the patients who received RP, some reports showed the effectiveness of the NLR as a predictor of biochemical recurrence [17, 20–22]. On the other hand, for the patients who have low-risk prostate cancer, the NLR was not a useful predictor for biochemical recurrence [23].

IHC was performed to detect CD66b-positive neutrophils and CD8-positive lymphocytes in RP specimens. However, there was no significant difference in the number of infiltrating CD66b-positive or CD8-positive cells between tissues from normal-appearing prostate and prostate cancer. Furthermore, no significant correlations between the neutrophil number, lymphocyte number, or their ratio and tumor characteristics (e.g., GS and pathological stage) or patient outcome were observed. A previous immunohistochemical study in esophageal squamous cell carcinoma specimens demonstrated that intratumoral neutrophils, CD8-positive lymphocytes, and their ratio, as seen in the NLR in CBCs, correlated with disease progression [24]. However, no attempts in other tissue specimens have been made to determine the role of the NLR in tumorigenesis or tumor progression as a biomarker.

Cho et al. reported that patients with an elevated NLR exhibit a relative lymphocyte-mediated immune response to malignancy, thereby worsening their prognosis and increasing the potential for tumor progression [25, 26]. The interaction between the tumor and host immune system promotes tumor cell proliferation, metastasis, and activation of the inflammatory cascade in the host, which further deteriorates the general condition of cancer patients [27].

There are several limitations associated with this study. First, the study was retrospective in nature and had a limited sample size, as we enrolled patients who obtained CBCs preoperatively from 73 constitutive patients in our institution. On the other hand, the pathological results of RP were often dependent on the surgical procedure; thus, to exclude this bias we obtained the data only from the cases that underwent RP by one surgeon (Yasuhide Miyoshi). Additionally, the sample size of this study was small compared with our previous study of prostate needle biopsies [13]. The NLR as a prognostic factor in solid malignancies was previously reported mainly in patients with advanced stages [17, 28, 29]. Because the patients who received RP had localized prostate cancer, the differences may be smaller. Finally, an immunohistochemical analysis of CD8 and CD66b was performed using another patient cohort.

In conclusion, in the current study, there was no association between NLR and biochemical failure after prostatectomy. Further investigation is needed to confirm our results.

## Figures and Tables

**Table 1:** Patients' characteristics.

	Number (%) or median (mean ± SD)
Age (years)	66 (66.0 ± 5.95)
Initial PSA (ng/mL)	8.5 (11.0 ± 6.5)
NCCN risk criteria	
Low	10 (13.7%)
Intermediate	35 (47.9%)
High	28 (38.4%)
NLR	1.85 (2.20 ± 1.04)
Gleason score	
≤6	12 (16.4%)
7	51 (69.8%)
≥8	10 (13.7%)
Pathological T stage	
2	51 (69.9%)
3	21 (28.8%)
4	1 (1.4%)
Lymph node metastasis	5 (6.8%)
Positive resection margin	27 (37.0%)
PSA failure	15 (20.5%)
Observation period	20.7 (25.9 ± 20.8)

**Table 2:** Clinicopathologic characteristics of prostate cancer patients used for immunohistochemical analysis.

	Number (%) or median (mean ± SD)
Number of patients	51
Age (years)	68 (65.1 ± 11.6)
Initial PSA (ng/mL)	12.8 (13.9 ± 7.1)
Gleason score	
≦6	10 (19.6%)
7	28 (54.9%)
≧8	13 (25.5%)
Pathological T stage	
2	30 (58.2%)
≧3	20 (39.2%)
